# Technical Quality of Root Canal Treatment Performed by Undergraduate Clinical Students of Isfahan Dental School 

**DOI:** 10.22037/iej.v13i1.18517

**Published:** 2018

**Authors:** Masoud Saatchi, Golshan Mohammadi, Armita Vali Sichani, Saba Moshkforoush

**Affiliations:** a *Dental Research Center, Department of Endodontics, Dental School, Isfahan University of Medical Sciences, Isfahan, Iran; *; b * Dental School, Isfahan University of Medical Sciences, Isfahan, Iran; *; c * Dental Material Research Center, Department of Endodontics, Dental School, Isfahan University of Medical Sciences. Isfahan, Iran; *; d * Dental Students’ Research Committee, Dental School, Isfahan University of Medical Sciences, Isfahan, Iran*

**Keywords:** Endodontics, Periapical Radiograph, Procedural Errors, Root Canal Treatment, Undergraduate Dental Student

## Abstract

**Introduction:**

The aim of the present study was to evaluate the radiographic quality of RCTs performed by undergraduate clinical students of Dental School of Isfahan University of Medical Sciences.

**Methods and Materials:**

In this cross sectional study, records and periapical radiographs of 1200 root filled teeth were randomly selected from the records of patients who had received RCTs in Dental School of Isfahan University of Medical Sciences from 2013 to 2015. After excluding 416 records, the final sample consisted of 784 root-treated teeth (1674 root canals). Two variables including the length and the density of the root fillings were examined. Moreover, the presence of ledge, foramen perforation, root perforation and fractured instruments were also evaluated as procedural errors. Descriptive statistics were used for expressing the frequencies of criteria and *chi* square test was used for comparing tooth types, tooth locations and academic level of students (*P*<0.05).

**Results:**

The frequency of root canals with acceptable filling was 54.1%. Overfilling was found in 11% of root canals, underfilling in 8.3% and inadequate density in 34.6%. No significant difference was found between the frequency of acceptable root fillings in the maxilla and mandible (*P*=0.072). More acceptable fillings were found in the root canals of premolars (61.3%) than molars (51.3%) (*P*=0.001). The frequency of procedural errors was 18.6%. Ledge was found in 12.5% of root canals, foramen perforation in 2%, root perforation in 2.4% and fractured instrument in 2%. Procedural errors were more frequent in the root canals of molars (22.5%) than the anterior teeth (12.3%) (*P*=0.003) and the premolars (9.5%) (*P*<0.001).

**Conclusion:**

Technical quality of RCTs performed by clinical students was not satisfactory and incidence of procedural errors was considerable.

## Introduction

Root canal treatment (RCT) is an important part of comprehensive dental care [1]. High prevalence of apical periodontitis in endodontically treated teeth, as reported by epidemiological studies [2, 3], reveals that the outcome of RCT in many populations is poor, which can elicit medical, economic and ethical consequences as a health care problem [4].

The outcome of primary endodontic treatment has been reported to be correlated with many factors [3, 5, 6]. One of these factors is the technical quality of RCT that could be measured by different methods [7-9], but usually and most commonly the evaluation method is radiographic [10]. It has been shown that the length of the root filling, relative to the radiographic apex, significantly affects the treatment outcomes [11]. Root fillings with adequate density are strongly associated with a lower risk of periapical disease [12]. In addition, procedural errors such as ledges, zip and elbow formations, fractured instruments and perforations may occur during RCT procedures [13]. These errors may result in compromised cleaning and shaping, leakage through root filling and infection of the periradicular tissues and can jeopardize the endodontic outcomes [13]. 

In the Dental School of Isfahan University of Medical Sciences, Endodontic curriculum includes theoretical, preclinical and clinical training. Preclinical training is presented as 2 courses during the third year of the undergraduate educational program. Each course includes 54 h of both lectures and practical training on extracted human teeth. During the preclinical practice, students are expected to complete RCTs on 4 anterior teeth, 4 premolars and 4 molars. Clinical training contains 3 courses of 68 h each and is presented during the fourth, fifth and sixth year. In the fourth year, students are required to perform RCTs on 6 single rooted teeth including anterior teeth and premolars. During the next clinical courses, fifth- year students are expected to perform RCTs on at least 2 molars and 2 premolars and sixth- year students are required to perform RCTs on at least 3 molars and 2 premolars.

Recent studies concerning technical quality of RCTs, show that undesirable quality is a common finding in RCTs performed by general dental practitioners and undergraduate dental students [1, 14-23]. Such studies can indicate inadequacies in clinical performance and can help decrease the undesirable outcomes by elevating educational programs [24].

Thus, the aim of the present study was to evaluate the technical quality of root canal treatments (quality of root fillings and incidence of procedural errors) performed by clinical undergraduate students in the Dental School of Isfahan University of Medical Sciences from 2013 to 2015.

## Materials and Methods

In this cross-sectional study, from the total records of 3960 patients who had undergone RCT in the Dental School of Isfahan University of Medical Sciences between 2013 and 2015, 1200 records were randomly selected. Four hundred and sixteen records were excluded and the final sample involved 784 endodontically treated teeth, consisting of 1674 canals.

Criteria for endodontic records to be included in the study consisted of being performed by fourth-, fifth- and sixth- year students during September 2013 to September 2015.

Exclusion criteria consisted of missing radiographs from the record and unreadable radiographs due to superimposition of adjacent structures, over or under exposure of the film, excessive elongation or foreshortening of the image and over or under development of the film.

The RCTs were accomplished according to the following protocol. The root canals were prepared by conventional hand files and obturated using cold gutta-percha lateral condensation technique. For root canal treatment of each tooth, 4 periapical radiographs (preoperative, working length determination, master cone and postoperative) were taken by the bisecting angle technique, using a dental radiography machine (DeGotzen, Roma, Italy) and E-speed #2 intraoral films (Primax, Berlin, Germany). Developing solutions (Champion Photochemistry International Ltd, UK) were used to possess the radiographs in a time-temperature technique. Endodontic Department’s academic stuff supervised all the treatment steps. The average academic stuff-to-student ratio was 1: 6 at the time of the study.

In order to evaluate the quality of each RCT, at least 3 periapical radiographs (PAR), including preoperative, working length determination and postoperative, were examined. Evaluations were made in a dark room under even illumination with 3× magnification. The PARs were mounted in a cardboard slit to exclude the extraneous light. Measurements were made using a transparent ruler of 0.5 mm accuracy. In cases in which the radiographic images had been taken with a change in horizontal angulation, it was supposed that they had been exposed with a mesial angulation. 

Two individual investigators (GM and AV) evaluated the PARs of each record. The results were compared and in case of disagreement a third investigator (MS) was asked to examine the records, and a final agreement was achieved.

Before the study, the investigators were calibrated and inter-examiner agreement was determined by evaluating 30 radiographic records that were not included in the study. For establishing intra-examiner agreement, each investigator re-evaluated the same radiographs after 2 weeks.

Evaluation of quality of RCTs was accomplished by examining the radiographic quality of root fillings and detection of the procedural errors. Each root canal was considered as the unit of evaluation.

The quality of root filling in each canal was categorized as acceptable and unacceptable according to the criteria used by Khabbaz *et al.* [19]:

1. Acceptable root filling: root filling ending 0‒2 mm from the radiographic apex without any visible voids in the filling mass or between the filling mass and root canal walls.
2. Unacceptable root filling:

- Overfilling: root filling extending beyond radiographic apex.
- Underfilling: root filling ending 2 mm short of the radiographic apex.
- Inadequate density: root filling with visible voids in the filling mass or between the filling mass and root canal walls on the final radiograph.

The criteria for the detection of procedural errors in this study were according to the criteria used by Khabbaz *et al.* [19]:

1. A ledge was identified if the root filling on the final radiograph did not follow the curvature of the main canal path on the working length radiograph.
2. Root perforations (including furcation perforation, strip perforation and lateral perforations of the root) were detected when extrusion of the filling materials was identified in any area of the root except the apical foramen.
3. Foramen perforation was diagnosed when the apical termination of the filled canal appeared as an elliptical shape transported to the outer wall.
4. Fractured instrument was detected by observing a part of instrument in the root canal or in periradicular area on the final radiograph.

Data were analyzed with SPSS software (SPSS version 21, IBM Inc., Chicago, USA). Descriptive analyzes were used for expressing frequencies of radiographic criteria of quality of RCTs. Pearson’s *chi* square test was used to compare the results among tooth types and locations and also academic year of students. Inter-examiner and intra-examiner agreements were evaluated using Cohen’s kappa (k) value. *P*-values less than 0.05 were considered statistically significant. 

**Table 1:** Acceptable and unacceptable root fillings according to tooth type and location

Tooth location	Tooth type	Total RF	Acceptable RF	Unacceptable RF*		
				Under filled	Over filled	Density problem
Maxilla	Anterior	119	69 (58%)	4 (3.4%)	19 (16%)	37 (31.1%)
	Premolar	268	174 (64.9%)	8 (3%)	30 (11.2%)	72 (26.9%)
	Molar	479	244 (50.9%)	47 (9.8%)	60(12.5%)	168 (35.1%)
	Total	866	487 (56.2%)^a^	59(6.8%)	109 (12.6%)	277(2%)
Mandible	Anterior	44	25 (56.8%)	3 (6.8%)	6 (13.6%)	16 (36.4%)
	Premolar	99	51 (51.5%)	6 (6.1%)	10 (10.1%)	40(40.4%)
	Molar	665	343 (51.6%)	71 (10.7%)	59 (8.9%)	247 (37.1%)
	Total	808	419 (51.9%)	80 (9.9%)	75 (9.3%)	303 (37.5%)
Total	Anterior	163	94 (57.7%) ^b^^, ^^c^	7 (4.3%)	25 (15.3%)	53 (32.5%)
	Premolar	367	225 (61.3%) ^d^	14 (3.8%)	40 (10.9%)	112 (30.5%)
	Molar	1144	587 (51.3%)	118 (10.3%)	119 (10.4%)	415 (36.3%)
	Total	1674	906 (54.1%)	139 (8.3%)	184 (11%)	580 (34.6%)

* (RF=Root Filling);

a No significant difference (P=0.072) between acceptable root fillings in the maxilla and mandible;

b No significant difference (P=0.430) between acceptable root fillings in anterior teeth and premolars;

c No significant difference (P=0.128) between acceptable root fillings in anterior teeth and molars;

d Significant difference (P=0.001) between acceptable root fillings in premolars and molars

**Table 2:** Acceptable and unacceptable root fillings according to academic year of students

Academic year	Total RF	Acceptable RF	Unacceptable RF*				
			Under filled		Over filled		Density problem
4th	311	173 (55.6%) ^a^^, ^^b^	13 (4.2%)	42 (13.5%)		110 (35.4%)	
5th	536	247 (46.1%) ^c^	69 (12.9%)	60 (11.2%)		217 (40.4%)	
6th	827	486 (58.8%)	57 (6.9%)	82 (9.9%)		253 (30.1%)	
Total	1674	906 (54.1%)	139 (8.3%)	184 (11%)		580 (34.6%)	

* (RF = Root Filling);

a Significant difference (P=0.007) between root fillings performed by fourth- and fifth-year students;

b No significant difference (P=0.339) between root fillings performed by fourth- and sixth-year students;

c Significant difference (P<0.001) between root fillings performed by fifth- and sixth-year students

**Table 3:** Distribution of procedural errors according to academic year of students

Academic year	Root canals treated	Total procedural errors	ledges	Foramen perforations	Root perforations	Separated instruments
**4th**	311	36 (11.6%)^a^^, ^^b^	22 (7.1%)	5 (1.6%)	7 (2.2%)	2 (0.6%)
**5th **	536	119 (22.2%)^c^	83 (15.5%)	13 (2.4%)	13 (2.4%)	13 (2.4%)
**6th **	827	157 (19%)	105 (12.7%)	16 (1.9%)	21 (2.5%)	19 (2.3%)
**Total**	1674	312 (18.6%)	210 (12.5%)	34 (2%)	41 (2.4%)	34 (2%)

a Significant difference (P<0.001) between root canals with procedural errors treated by fourth- and fifth-year students;

b Significant difference (P=0.003) between root canals with procedural errors treated by fourth- and sixth-year students;

c No significant difference (P=0.149) between root canals with procedural errors treated by fifth- and sixth-year students

## Results

Of 1200 collected records of endodontically treated teeth, 416 cases (34.7%) were excluded and 784 teeth, consisting of 1674 root canals, were evaluated; 52% of the treated root canals were in the maxilla and 48% were in the mandible. The root canals of molars comprised the most frequently treated root canals (68%), followed by premolars (22%) and anterior teeth (10%). Sixth-year students performed RCTs on 49% of the root canals. The fifth- and fourth-year students treated 32% and 19% of the root canals, respectively. The anterior teeth and premolars constituted the teeth treated by fourth-year students while fifth- and sixth-year students treated premolars and molars.

Technical quality of root fillings: According to the length and density, acceptable fillings were found in 54.1% of the root canals. In the maxilla, 56.2% and in the mandible, 51.9% of the root fillings were acceptable. The rate of acceptable root fillings was not significantly different between the two arches (*P*=0.072). Among tooth types, the root canals of molars exhibited a lower ratio of acceptable root canal fillings (51.3%) compared to premolars (61.3%) (*P*=0.001). The rate of acceptable fillings in the anterior teeth (57.7%) was not significantly different from molars (*P*=0.128) and premolars (*P*=0.430). Inadequate density, overfilling and underfilling were found in 34.6%, 11% and 8.3% of root canals, respectively. In both arches inadequate density was the most common cause for unacceptable filling. In the maxilla overfilling was the second frequent cause for unacceptable filling, followed by underfilling. However, underfilling was the second common cause for unacceptable fillings in the mandible and overfilling was the least frequent cause (Table 1).

Of the root canals treated by fifth-year students, 46.1% had acceptable fillings, which is significantly lower than the canals treated by fourth-year (55.6%) (*P*=0.007) and sixth-year students (58.8%) (*P*<0.001). No significant difference was found in the frequencies of acceptable fillings performed by fourth- and sixth-year students (*P*=0.339) (Table 2).

Procedural errors: Procedural errors were found in 18.6% of endodontically treated root canals. The incidence of procedural errors between the fifth-year (22.2%) and sixth-year students (19%) was not significantly different (*P*=0.149); fourth-year students had created less procedural errors (11.6%) than fifth-year (*P*<0.001) and sixth-year students (*P*=0.003).

Ledge was the most frequent procedural error and was identified in 12.5% of endodontically treated root canals. Foramen perforation, root perforation and fractured instrument were detected in 2%, 2.4% and 2% of the root canals, respectively (Table 3).

The incidence of procedural errors was not significantly different in the root canals of anterior teeth (12.3%) and premolars (9.5%) (*P*=0.341). These errors were significantly more frequent in the root canals of molars (22.5%) compared to anterior teeth (*P*=0.003) and premolars (*P*<0.001) (Table 4).

The k-value for inter-examiner agreement was 0.87 for detection of acceptable root fillings and 0.81 for identification of RCTs without procedural errors. For intra-examiner agreement k-values for detection of acceptable root fillings and identification of RCTs without procedural errors were 0.93 and 0.87 for the first and 0.84 and 0.81 for the second investigator, respectively. 

## Discussion

This study was designed to evaluate the technical quality of root canal treatments accomplished by undergraduate students in the Dental School of Isfahan University of Medical Sciences. Periapical radiographs taken during and after the RCT procedures were used for this investigation. Root fillings were considered acceptable if terminated 0‒2 mm short of the radiographic apex and had no voids. These criteria are extensively documented to be associated with improved periapical health [3, 10, 12].

In order to limit inter-examiner and intra-examiner discrepancies, the radiographic criteria were strictly defined and two investigators were calibrated before the study. It has been reported that great variations could exist between investigators regarding assessment of the technical quality of RCTs [17]. In the present study, the k-value of 0.87 for detection of acceptable root fillings and 0.81 for identification of RCTs without procedural errors exhibited good agreement between the investigators. In addition, k-values for intra-examiner agreement were found to be greater than 0.81, which shows the reliability of each investigator.

Acceptable root fillings according to the length and density were found in 54.1% of the root canals. Among the studies about the quality of RCTs performed by undergraduate students, the frequency of acceptable fillings in the current study is comparable to the findings of some studies [17, 19], higher than other studies [18, 24] and less than one study [25]. This may be due to the differences in the sample selection and criteria used by these studies.

**Table 4:** Distribution of procedural errors according to tooth type

Tooth Type	Root canals treated	Procedural errors
Anterior	163	20 (12.3%) ^a^^, ^^b^
Premolars	367	35 (9.5%) ^c^
Molars	1144	257 (22.5%)
Total	1674	312 (18.6%)

a No significant difference (P=0.341) between root canals with procedural errors in anterior teeth and premolars;

b Significant difference (P=0.003) between root canals with procedural errors in anterior teeth and molars;

c Significant difference (P<0.001) between root canals with procedural errors in premolars and molars

In the present study, inadequate density was the most common cause for unqualified root fillings. This is consistent with the findings of Balto *et al.* [14]. It is believed that lateral condensation technique with gutta-percha might lead to voids in root canals with insufficient flaring [26] .

Adequate root canal fillings in molars were less than the premolars, consistent with the findings reported by Er *et al.* [18] and Khabbaz *et al.* [19], who also reported a lower quality of root fillings in molars. This might be explained by the posterior position and complex anatomy of these teeth. However, no significant difference was found between the quality of maxillary and mandibular root fillings. 

Fifth-year students had performed more unacceptable root fillings than the fourth- and sixth- year students. This can be explained by considering the fact that in the Dental School of Isfahan University of Medical Sciences, the undergraduate students’ first clinical encounter with molars takes place in their fifth year of study. Fourth- year students who perform RCTs only on single rooted teeth, accomplished 55.6% of acceptable root fillings. In the study of Lynch and Burke [25] that also investigated single rooted teeth, 63% of root fillings were acceptable. The difference could be due to the size of the sample and level of the students.

Procedural errors were detected in 18.6% of the root canals. These errors were more frequent in molars. Khabbaz *et al.* [19] and Balto *et al.* [14] also reported a high prevalence of procedural errors in molars. This might be explained by curved and narrow root canals of molars, which makes them challenging for undergraduate students [27]. Furthermore, fifth- and sixth-year students had created more procedural errors than fourth-year students. This is because fourth-year students only performed RCTs on anterior teeth and premolars which are less challenging than molars. There was no significant difference between the incidence of procedural errors created by fifth- and sixth-year students. Sixth-year students are more experienced in treating molar teeth but they also try performing RCTs on more difficult cases than fifth-year students. Therefore, the rate of procedural errors was not significantly different between these two educational courses.

In this study, ledge was found to be the most frequent procedural error and was detected in 12.5% of the root canals. This finding is consistent with the frequency of ledge in the study by Khabbaz *et al.* [19] and is less than that in a study by Eleftheriadis and Lambrianidis [17]. It has been shown that stainless steel hand files used by inexperienced undergraduate students could increase the incidence of ledge and other procedural errors [17].

Radiographic images cannot illustrate all the procedural errors. For instance, over instrumentation which drives pulpal fragments and microorganisms beyond the apex into the periapical tissues can only be radiographically diagnosed when it is followed by extrusion of filling material from the apex. The use of bisecting-angle technique for taking periapical radiographs results in less accuracy in determining the root canal length compared to the parallel technique [17]. It has also been shown that using only one orthoradial radiographic image for assessing the adaptation of the filling material to the root canal walls is not reliable. This adaptation has to be further investigated with at least one extra radiography of distal or mesial angulation in order to obtain a more realistic estimate of the density of root canal filling [28]. Although radiographic quality of RCT is a significant determinant in predicting the outcomes of primary endodontic treatment, the radiographic images cannot reflect the general quality of treatment. Moreover, application of antiseptic and aseptic techniques, materials used and microbial condition of the root canal are the predicting factors which are not investigated in radiographic studies. 

In the Dental School of Isfahan University of Medical Sciences, passive step-back preparation and cold lateral condensation techniques are taught to undergraduate dental students. It has been shown that use of rotary Ni-Ti instruments provides better canal shaping, reduces the procedural errors [29] and is taught in undergraduate curriculum in some dental schools [15, 21].

According to many studies, insufficient time is allocated to clinical and preclinical training, and the academic stuff-to-student ratio, anxiety, and evaluation methods have been reported as reasons for the low quality of RCTs in university clinics [14, 19, 24, 30, 31]. It appears that enhancing the time allocated to clinical training and increasing stuff-to-student ratio can lead to improvements in the quality of RCTs performed by undergraduate students.

## Conclusions

According to the results of this study, technical quality of RCTs performed by clinical students was not satisfactory and incidence of procedural errors was considerable. Therefore, it seems that it is necessary to revise endodontic educational programs in order to improve the quality of root canal treatments.

## References

[1] Boucher Y, Matossian L, Rilliard F, Machtou P (2002). Radiographic evaluation of the prevalence and technical quality of root canal treatment in a French subpopulation. Int Endod J.

[2] Asgary S, Shadman B, Ghalamkarpour Z, Shahravan A, Ghoddusi J, Bagherpour A, Akbarzadeh Baghban A, Hashemipour M, Ghasemian Pour M (2010). Periapical status and quality of root canal fillings and coronal restorations in iranian population. Iran Endod J.

[3] Pak JG, Fayazi S, White SN (2012). Prevalence of periapical radiolucency and root canal treatment: a systematic review of cross-sectional studies. J Endod.

[4] Santos SM, Soares JA, Cesar CA, Brito-Junior M, Moreira AN, Magalhaes CS (2010). Radiographic quality of root canal fillings performed in a postgraduate program in endodontics. Braz Dent J.

[5] Craveiro MA, Fontana CE, de Martin AS, Bueno CE (2015). Influence of coronal restoration and root canal filling quality on periapical status: clinical and radiographic evaluation. J Endod.

[6] Song M, Park M, Lee C-Y, Kim E (2014). Periapical status related to the quality of coronal restorations and root fillings in a korean population. J Endod.

[7] Alves RAA, Souza JB, Gonçalves Alencar AH, Pécora JD, Estrela C (2013). Detection of procedural errors with stainless steel and niti instruments by undergraduate students using conventional radiograph and cone beam computed tomography. Iran Endod J.

[8] Naseri M, Kangarlou A, Khavid A, Goodini M (2013). Evaluation of the quality of four root canal obturation techniques using micro-computed tomography. Iran Endod J.

[9] Shantiaee Y, Maziar F, Dianat O, Mahjour F (2011). Comparing microleakage in root canals obturated with nanosilver coated gutta-percha to standard gutta-percha by two different methods. Iran Endod J.

[10] Ng YL, Mann V, Rahbaran S, Lewsey J, Gulabivala K (2008). Outcome of primary root canal treatment: systematic review of the literature–Part 2 Influence of clinical factors. Int Endod J.

[11] Moreno JO, Alves FR, Goncalves LS, Martinez AM, Rocas IN, Siqueira JF Jr (2013). Periradicular status and quality of root canal fillings and coronal restorations in an urban Colombian population. J Endod.

[12] Chugal NM, Clive JM, Spangberg LS (2003). Endodontic infection: some biologic and treatment factors associated with outcome. Oral Surg Oral Med Oral Pathol Oral Radiol Endod.

[13] Peters OA (2004). Current challenges and concepts in the preparation of root canal systems: a review. J Endod.

[14] Balto H, Al Khalifah S, Al Mugairin S, Al Deeb M, Al-Madi E (2010). Technical quality of root fillings performed by undergraduate students in Saudi Arabia. Int Endod J.

[15] Donnelly A, Coffey D, Duncan HF (2017). A re-audit of the technical quality of undergraduate root canal treatment after the introduction of new technology and teaching practices. Int Endod J.

[16] Dugas NN, Lawrence HP, Teplitsky PE, Pharoah MJ, Friedman S (2003). Periapical health and treatment quality assessment of root-filled teeth in two Canadian populations. Int Endod J.

[17] Eleftheriadis GI, Lambrianidis TP (2005). Technical quality of root canal treatment and detection of iatrogenic errors in an undergraduate dental clinic. Int Endod J.

[18] Er O, Sagsen B, Maden M, Cinar S, Kahraman Y (2006). Radiographic technical quality of root fillings performed by dental students in Turkey. Int Endod J.

[19] Khabbaz MG, Protogerou E, Douka E (2010). Radiographic quality of root fillings performed by undergraduate students. Int Endod J.

[20] Moradi S, Gharechahi M (2014). Quality of root canal obturation performed by senior undergraduate dental students. Iran Endod J.

[21] Moussa‐Badran S, Roy B, Bessart du Parc A, Bruyant M, Lefevre B, Maurin J (2008). Technical quality of root fillings performed by dental students at the dental teaching centre in Reims, France. Int Endod J.

[22] Ribeiro DM, Reus JC, Felippe WT, Pacheco-Pereira C, Dutra KL, Santos JN, Porporatti AL, De Luca Canto G (2017). Technical quality of root canal treatment performed by undergraduate students using hand instrumentation: a meta-analysis. Int Endod J.

[23] Unal GC, Kececi AD, Kaya BU, Tac AG (2011). Quality of root canal fillings performed by undergraduate dental students. Eur J Dent.

[24] Eskandarloo A, Karkehabadi H, Hoseini Hashemi SZ, Ahmadi M, Hendi SS (2017). Radiographic quality of root canal obturation performed by fifth year students of hamadan dental school. Iran Endod J.

[25] Lynch C, Burke F (2006). Quality of root canal fillings performed by undergraduate dental students on single‐rooted teeth. Eur J Dent Educ.

[26] Ho ESS, Chang JWW, Cheung GSP (2016). Quality of root canal fillings using three gutta-percha obturation techniques. Restorative dentistry & endodontics.

[27] Yavari H, Samiei M, Shahi S, Borna Z, Abdollahi AA, Ghiasvand N, Shariati G (2015). Radiographic evaluation of root canal fillings accomplished by undergraduate dental students. Iran Endod J.

[28] Eckerbom M, Magnusson T (1997). Evaluation of technical quality of endodontic treatment--reliability of intraoral radiographs. Endod Dent Traumatol.

[29] Dahlstrom L, Molander A, Reit C (2011). Introducing nickel-titanium rotary instrumentation in a public dental service: the long-term effect on root filling quality. Oral Surg Oral Med Oral Pathol Oral Radiol Endod.

[30] Alsulaimani RS, Al-Manei KK, S AA, AlAqeely RS, S AMA-S, E MA-M (2015). Effects of clinical training and case difficulty on the radiographic quality of root canal fillings performed by dental students in saudi arabia. Iran Endod J.

[31] Saatchi M, Abtahi M, Mohammadi G, Mirdamadi M, Binandeh ES (2015). The prevalence of dental anxiety and fear in patients referred to Isfahan Dental School, Iran. Dent Res J (Isfahan).

